# Be aware of non-specific presentation of pulmonary embolism: a case report

**DOI:** 10.1186/s12872-023-03096-z

**Published:** 2023-02-10

**Authors:** Hang Li, Qinghai Dai, Lingfeng Shu, Dongyi Yang, Tao Wu

**Affiliations:** 1 The First Clinical Medical College, Henan University of Traditional Chinese Medicine, Zhengzhou, 450000 Henan China; 2 Department of Intervention, The First Affiliated Hospital of Henan University of Traditional Chinese Medicine, Zhengzhou, 450000 Henan China

**Keywords:** syncope, Non-specific, Submassive, Pulmonary embolism, Case report

## Abstract

**Background:**

The early diagnosis of non-specific presentation of pulmonary embolism (PE) is difficult because the symptoms are non-specific and varied.

**Case presentation:**

A 69-year-old female patient had syncope accompanied by gait disturbance, without obvious inducement. The patient was initially suspected to have cerebral infarction, but the symptoms did not improve and myocardial markers increased after two days of symptomatic treatment for myocardial infarction. Hence, PE was suspected and computed tomography pulmonary angiography (CTPA) examination confirmed the diagnosis. CTPA showed multiple emboli in pulmonary artery and its branches, so high-risk PE was diagnosed. Intravenous thrombolysis was administered, and pulmonary CTA showed a significant reduction of emboli in pulmonary artery and its left and right branches.

**Conclusion:**

This case report highlights the importance of improving the clinical awareness about non-specific presentation of PE and avoiding misdiagnosis or missed diagnosis.

## Background

Pulmonary embolism (PE) is a common and fatal disease that should be diagnosed and treated promptly [1]. PE is usually caused by an occlusion of the pulmonary artery or its branches [2]. The clinical manifestations of PE vary from mild asymptomatic to chest tightness, chest pain, hemoptysis, severe hemodynamic decompensation, and even sudden death [3]. Early diagnosis of PE is occasionally difficult because its symptoms are non-specific and diverse. Here, we reported a patient with non-specific presentation of pulmonary embolism who complained of recurrent syncope.

## Case presentation

A 69-year-old female had syncope accompanied by gait disturbance, without obvious inducement at home on May 5, 2021. She had no chest distress, chest pain, dyspnea or other symptoms. She had a history of hypertension, the highest blood pressure was 160/90 mmHg and took nifedipine 20 mg po qd. She suffered from cerebral infarction one year ago, with no obvious sequelae, and no history of other diseases.

Admission signs: Heart rate 68 per minute, blood pressure 146/64 mmHg, vesicular breath sounds clear by auscultation, no rhonchi, no crackle, no abnormality in visual examination, palpation, percussion and auscultation of the heart, no edema in lower limbs, no abnormality in nervous system examination. Electrocardiogram (ECG) suggested T wave change of anterior wall. High-resolution CT of lung showed multiple fibrous cords in both lungs, without specificity of PE. Magnetic Resonance Angiography showed suspected small aneurysm of left posterior cerebral artery, without cerebrovascular stenosis. Ultrasound showed that EF was 60%, D-dimer was 0.44 µg/ml (0–0.5 µg/ml); BNP was 46 pg/ml (0.00–100 pg/ml), Tn was 0.12 ng/ml (0.00–0.04 ng/ml). The patient was given Citicoline sodium injection to nourish nerves, Shuxuetong injection to promote blood circulation and remove blood stasis, Uricolin injection to promote collateral circulation, and aspirin for anti-platelet aggregation.

On May 8, 2021, the patient developed syncope again, accompanied by palpitation. ECG showed changes of ST-T segments of inferior wall, anterior side wall and posterior wall, C-reactive protein was 5.9 mg/L (0.2–4 mg/L), D-dimer was 0.46 µg/ml, BNP was 70 pg/ml, Tn was 0.12 ng/ml, and creatine kinase isozyme was 2.6 ng/mL (0.6–6.3 ng/mL). Hence, myocardial infarction was considered, and other examinations were normal. Enoxaparin 0.55 ml sc bid was given to improve myocardial ischemia, and the changes were closely observed.

On May 9, 2021, the patient developed shock, blood pressure was 87/60 mmHg. ECG showed the ST-T segments of the lower and front side walls, but no obvious change compared with before. D-dimer was 6.19 µg/ml, creatine kinase isozyme was 9.4 ng/mL, Tn was 0.97 ng/ml, and myocardial markers did not conform to ACS, so the diagnosis of coronary heart disease was excluded. Bedside color Doppler ultrasound indicated EF 65%, pulmonary hypertension. Venous blood vessel examination of both lower limbs showed inter-muscular venous thrombosis of both lower legs, indicative of possible PE.

**Fig. 1:**
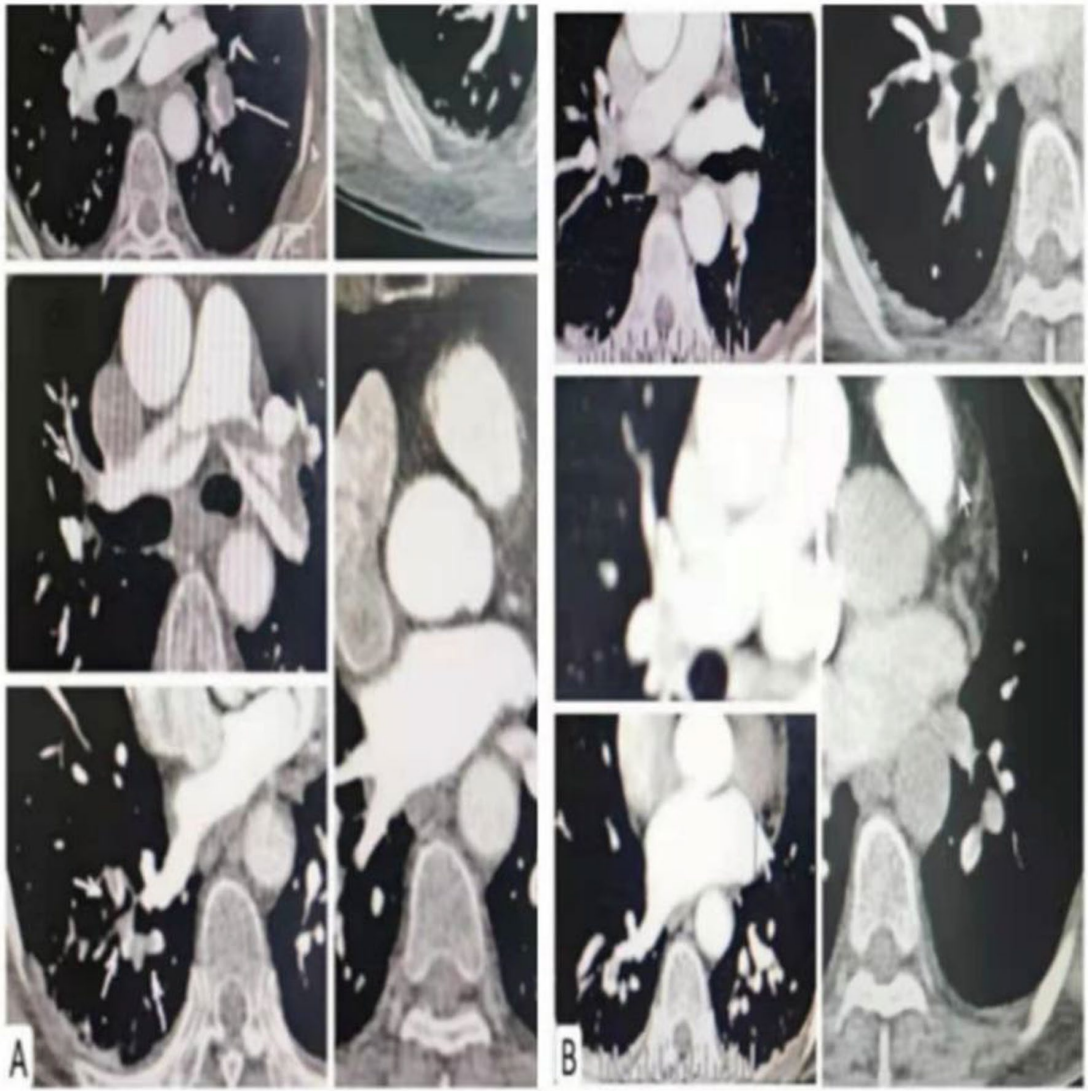
**A** Multiple emboli were seen in the main pulmonary artery and its left and right branches. **B** Pulmonary emboli had disappeared

**Fig. 2:**
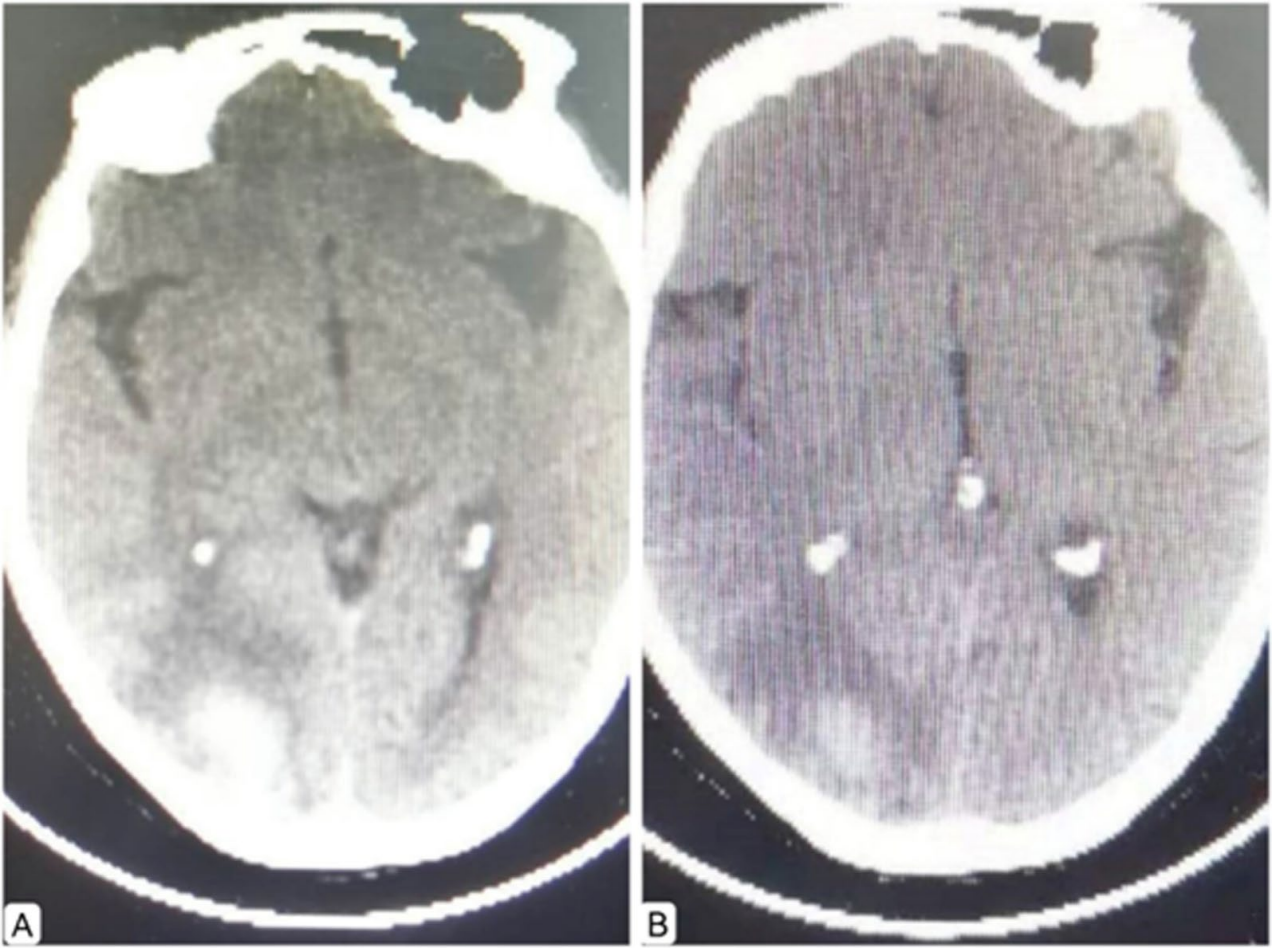
**A** Bleeding was seen in the right occipital lobe. **B** The volume of bleeding in the right occipital lobe was significantly reduced

CTPA showed multiple emboli in pulmonary artery and its branches (Fig. 1A), so high-risk PE was diagnosed. Intravenous thrombolysis was proposed, rt-PA 50 mg/50 mL was continuously given with syringe pumps within 120 min. Thereafter, pulmonary CTA showed a significant reduction of emboli in pulmonary artery and its left and right branches (Fig. 1B). Rivaroxaban po was continued for anti-coagulation. On May 16, 2021, the patient was discharged without recurrence of symptoms. One week later, the patient suffered a sudden cerebral hemorrhage (Fig. 2A). Delayed cerebral hemorrhage caused by intravenous thrombolysis was considered. The patient’s condition improved after administration of mannitol to lower intracranial pressure, and rivaroxaban tablets were discontinued (Fig. 2B).

## Conclusion

PE is the third most common cause of cardiovascular death worldwide, after ischemic stroke and heart attack. However, the misdiagnosis rate of PE remains very high, which seriously affects prognosis [4, 5]. PE may present typical features, such as dyspnea and pleuritic chest pain, or less typical, such as latent dyspnea for days to weeks, or relatively few respiratory symptoms, and the less typical symptoms are relatively difficult to diagnose [6]. In this case, the patient complained of syncope accompanied by gait disturbance, without chest tightness, chest pain, dyspnea and other symptoms. The patient was initially suspected to have cerebral infarction, but the symptoms did not improve after two days of symptomatic treatment, and myocardial markers increased. After two days of treatment for myocardial infarction, the symptoms did not improve, so PE was suspected and CTPA examination confirmed the diagnosis.

For patients with submissive PE, anticoagulant therapy is recommended, but blood pressure monitoring is necessary. Once hemodynamic decompensation is achieved, intravenous thrombolysis should be given in time [7]. The present case showed that timely intravenous thrombolysis can achieve a good prognosis. If not, catheter-directed thrombolytic therapies, mechanical thrombolysis, or transcatheter thrombus aspiration can be chosen [8].

## Data Availability

The datasets used and/or analysed during the current study are available from the corresponding author on reasonable request.

## Abbreviations

PE: Pulmonary embolism
CTPA: Computed Tomography Pulmonary Angiography
